# Late repair of tetralogy of Fallot is associated with increased aortic stiffness: a retrospective CMR cohort study

**DOI:** 10.1186/1532-429X-15-S1-O39

**Published:** 2013-01-30

**Authors:** Jason Christensen, Janet E Donohue, Sunkyung Yu, Jimmy C Lu, Maryam Ghadimi Mahani, Prachi Agarwal, Adam L Dorfman

**Affiliations:** 1 Pediatric Cardiology, University of Michigan, C.S. Mott Children's Hospital, Ann Arbor, MI, USA

## Background

Several studies report abnormal aortic properties in congenital heart disease patients, possibly worsening risk for aneurysm formation. We sought to describe patient characteristics and Cardiovascular Magnetic Resonance (CMR) parameters associated with worsened aortic strain and stiffness in a single center retrospective chart review of tetralogy of Fallot (TOF) patients undergoing CMR.

## Methods

227 TOF patients underwent CMR from 2007-2011 at the University of Michigan Health System. Exclusion criteria were: exposure to sedation, history of aortic stenosis, coarctation or prior aortic root replacement, surgical repair after 20 years of age, limited protocol or inadequate image quality for necessary measurements. Patient anthropomorphic measurements, clinical history, standard CMR measurements, aortic pulse wave velocity (PWV) and aortic strain data were collected, with strain defined as: (Areamin-Areamax)/(Areamin)x100; Areamax and Areamin are the maximal and minimal lumen areas (mm2) of the ascending aorta and PWV (m/s) calculated by the aortic path length between ascending and descending aorta/ transit time of systolic wave front. Correlation between aortic stiffness or strain and multiple parameters was examined via Pearson and Spearman correlation coefficients, two-sided t-test and Wilcoxon rank sum. Candidate variables were evaluated in a multivariate regression model for strength of association.

## Results

124 patients were included. Mean age at repair was 1.9 years and mean age at CMR was 24.6 years. 55 patients (44%) had a history of shunt placement. 29 patients (23.4%) were on anti-hypertensive or anti-arrhythmic medications. Mean LV ejection fraction was 54 ± 7.5%. Increased aortic stiffness was associated with age at repair (r=0.3, p=0.001), cross-sectional ascending aortic area (r=0.34, p=0.0001), aortic regurgitation (r=0.26, p=0.003) and history of shunt placement (p=0.01). In a multivariate model controlling for use of cardiac medications and history of shunt placement, the log transformed age at repair and increasing ascending aortic cross-sectional area were predictive of aortic stiffness (t-value=2.07, Rad2=0.11, p-value=0.04; t-value=2.9, Rad2=0.15, P-value=0.005, respectively). Decreased aortic strain was also associated with age at TOF repair (r=-0.5, p<0.001), cross-sectional ascending aortic area (r=-0.43, p<0.0001), aortic regurgitation (r=-0.45, p=<0.0001) and history of shunt placement (p=0.01). LV ejection fraction did not correlate with aortic parameters.

## Conclusions

Increasing age at TOF repair and cross-sectional area of the ascending aorta correlated with worsened aortic stiffness and strain and were independently associated with worsened aortic stiffness on multivariate regression models. Early repair may decrease mechanisms of aortic pathology that promote aneurysm formation, a treatable morbidity that may become more important as this population ages.

## Funding

N/A

**Figure 1:**
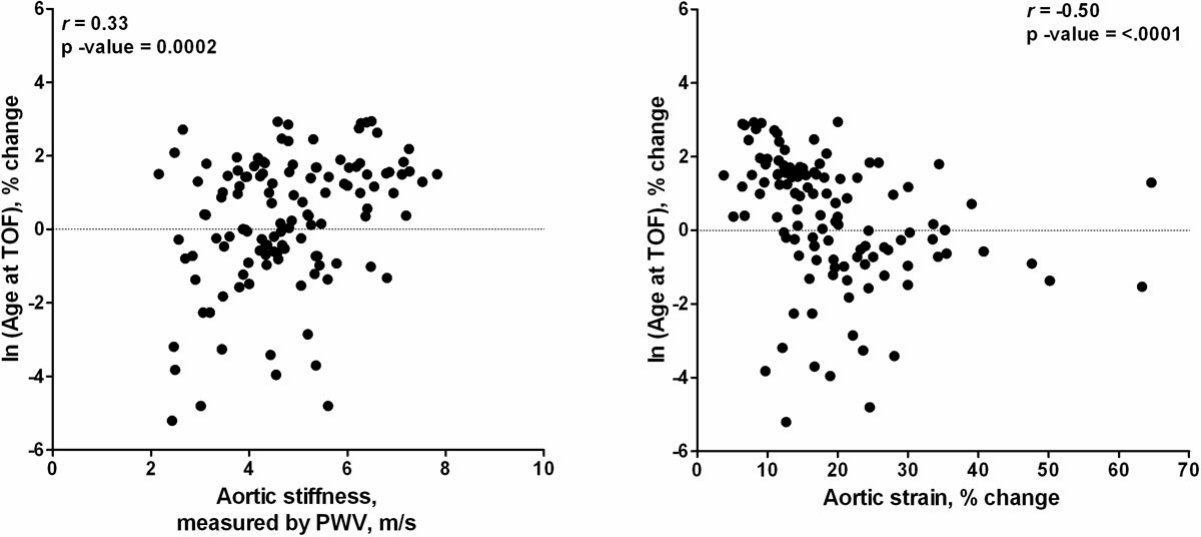
Aortic stiffness and strain vs. log transformed age at TOF repair

